# Supplementary material for: Preliminary Study on the Differences in Hydrocarbons Between Phosphine-Susceptible and -Resistant Strains of Rhyzopertha dominica (Fabricius) and Tribolium castaneum (Herbst) Using Direct Immersion Solid-Phase Microextraction Coupled with GC-MS
Source: Molecules. 2020 Mar 29;25(7):1565. doi: 10.3390/molecules25071565 (PMC7180957; doi:10.3390/molecules25071565)
Supplement: Supplementary file 1 [file molecules-25-01565-s001.pdf]

**Suplemantary materials**

***Table S1.*** Extracted and identified hydrocarbon compounds from cuticle layer and homogenised body of *T. castaneum.*

| No | ID | Compound name | NIST RI | Calcu-lated RI | GC-MS response ($10^5$) ± SD, n=4 Cuticular hydrocarbons | | Fold change (A/B) | p value ($10^{-3}$) | GC-MS response ($10^5$) ± SD, n=4 Homogenised body hydrocarbons | | Fold change (A/B) | p value ($10^{-3}$) |
|---|---|---|---|---|---|---|---|---|---|---|---|---|
| | | | | | Resistant (A) | Susceptible (B) | | | Resistant (A) | Susceptible (B) | | |
| **1** | 16.28_154 | 1-Pentadecene | 1502 | 1504 | 1639±50 | 1017±161 | 1.61 | 0.31 | 1451±111 | 1172±113 | 1.24 | 7.90 |
| **2** | 31.48_253 | Unknown1 | - | 2505 | 67±17 | 8±1 | 8.24 | 0.40 | 25±5 | 4±1 | 6.31 | 12.00 |
| **3** | 33.32_238 | Pentacosane | 2500 | 2515 | 195±8 | 122.07±24 | 1.60 | 1.10 | 110±15 | 53±9 | 2.09 | 0.59 |
| **4** | 33.89_266 | Hexacosane | 2600 | 2612 | 17±1 | 18±2 | 0.93 | 230.00 | 13±2 | 9±1 | 1.46 | 6.90 |
| **5** | 34.06_267 | Unknown2 | - | 2618 | 123±8 | 72±20 | 1.72 | 2.90 | 78±5 | 42±5 | 1.87 | 0.057 |
| **6** | 34.27_294 | 2-methylhexacosane | 2661 | 2684 | 7±1 | 6±1 | 1.29 | 100.00 | 8.78±1.69 | 4±0.4 | 2.18 | 1.30 |
| **7** | 34.73_296 | 13-methylheptacosane | 2731 | 2741 | 509±63 | 236±43 | 2.16 | 0.37 | 235±67 | 274±46 | 0.86 | 370.00 |
| **8** | 34.82_309 | 11-methylheptacosane | 2734 | 2750 | 2399±57 | 1799±363 | 1.33 | 17.00 | 1609±133 | 1024±122 | 1.57 | 0.64 |
| **9** | 35.02_336 | 2-methylheptacosane | 2762 | 2766 | 50±7 | 29±4 | 1.71 | 1.90 | 31±2 | 11±2 | 2.76 | 0.013 |
| **10** | 35.58_337 | 3-methylheptacosane | 2773 | 2771 | 2180±156 | 1584±352 | 1.38 | 21.00 | 1427±116 | 839±87 | 1.70 | 0.18 |
| **11** | 35.87_323 | Octacosane | 2800 | 2815 | 634±29 | 393±96 | 1.62 | 2.900 | 323±18 | 254±37 | 1.27 | 1.00 |
| **12** | 36.28_351 | 3-methyloctacosane | 2872 | 2849 | 453±12 | 369±57 | 1.23 | 28.00 | 217±25 | 128±18 | 1.69 | 1.20 |
| **13** | 36.60_365 | Nonacosane | 2900 | 2902 | 134±10 | 80±17 | 1.67 | 1.50 | 51±6 | 27±3 | 1.88 | 0.58 |
| **14** | 36.85_324 | Unknown3 | - | 2908 | 162±13 | 95±21 | 1.70 | 1.70 | 74±8 | 38±5 | 1.96 | 0.28 |
| **15** | 37.08_394 | Unknown4 | - | 2911 | 91±4 | 62±14 | 1.45 | 8.30 | 36±7 | 15±2 | 2.44 | 1.20 |
| **16** | 37.49_378 | Unknown5 | - | 2917 | 427±40 | 346±50 | 1.23 | 45.00 | 200±34 | 364±67 | 0.55 | 4.40 |
| **17** | 37.58_379 | 13-methylnonacosane | 2930 | 2927 | 584±47 | 501±170 | 1.17 | 380.00 | 233±36 | 128±18 | 1.81 | 2.00 |
| **18** | 37.92_393 | 11-methylnonacosane | 2939 | 2950 | 188±31 | 149±52 | 1.26 | 250.00 | 66±13 | 35±6 | 1.87 | 1.60 |
| **19** | 38.13_421 | Nonacosane, 2-methyl- | 2962 | 2961 | 409±28 | 282±69 | 1.45 | 14.00 | 156±16 | 95±13 | 1.65 | 0.64 |
| **20** | 38.44_395 | 3-methylnonacosane | 2974 | 2973 | 87±6 | 39±8 | 2.21 | 0.068 | 40±6 | 18±2 | 2.17 | 0.53 |
| **21** | 39.64_239 | Triacontane | 3000 | 3003 | 35±5 | 2±5 | 1.44 | 27.00 | 16±1 | 10±1 | 1.59 | 0.17 |
| **22** | 42.37_449 | Dotriacontane | 3200 | 3203 | 41±2 | 37±4 | 1.12 | 75.00 | 12±2 | 7±1 | 1.70 | 4.60 |

The list consists of only the compounds that were identified; some compounds may present on the GC-MS chromatogram but are not presented due to the lack of the identification; ID includes the retention time and mass to charge ratio that used to identify the chemical; "Unknown" features are compounds that were identified as hydrocarbons by NIST database, but were reported as unknown because of the big difference between the NIST RI and the calculated RI (> 30 difference); RT=retention time; NIST RI=retention indices acquired from National Institute of Standards and Technology database (NIST); Calculated RI=retention indices calculated using n-alkane standard C7-C40; Fold changes obtained from dividing GC-MS response (areas) of resistant insects by the GC-MS response (areas) of susceptible insects; p values were generated by t-tests as ($p \leq 0.05$, n=4); ND=not detected.

***Table S2.*** Extracted and identified hydrocarbon compounds from cuticle layer and homogenised body of *R. dominica*.

| NO | ID | Compound name | NIST RI | Calcula-ted RI | GC-MS response ($10^5$) ±SD, n=4 Cuticular hydrocarbons | | Fold change (A/B) | p value ($10^{-3}$) | GC-MS response ($10^5$) ±SD, n=4 Homogenised body hydrocarbons | | Fold change (C/D) | p value ($10^{-3}$) |
|---|---|---|---|---|---|---|---|---|---|---|---|---|
| | | | | | Resistant (A) | Susceptible (B) | | | Resistant (C) | Susceptible (D) | | |
| **1** | 31.46_253 | Unknown1 | - | 2515 | 35±9 | 12±3 | 2.77 | 6.90 | 15±4 | ND | NA | 0.28 |
| **2** | 31.94_281 | 11-methylpentacosane | 2535 | 2555 | 330±61 | 6±1 | 59.17 | 0.044 | 7±1 | 2±0.3 | 3.65 | 0.014 |
| **3** | 32.91_295 | Unknown2 | - | 2628 | 27±8 | 17±3 | 1.64 | 80.00 | 11±2 | ND | NA | 100.00 |
| **4** | 34.41_296 | 13-methylheptacosane | 2731 | 2741 | 156±18 | 260±41 | 0.60 | 100.00 | 236±92 | 143±35 | 1.65 | 0.14 |
| **5** | 34.84_336 | 2-methylheptacosane | 2762 | 2766 | 373±87 | 96±24 | 3.91 | 1.40 | 111±10 | 33±3 | 3.38 | 0.0046 |
| **6** | 35.34_337 | 3-methylheptacosane | 2773 | 2771 | 73±15 | 70±10 | 1.03 | 0.16 | 60±9 | 35±11 | 1.70 | 13.00 |
| **7** | 35.72_323 | Octacosane | 2800 | 2815 | 73±15 | 57±10 | 1.28 | 120.00 | 33±9 | 31±6 | 1.07 | 700.00 |
| **8** | 37.10_394 | Unknown3 | - | 2912 | 306±40 | 306±52 | 1.00 | 110.00 | 147±27 | 220±31 | 0.67 | 12.00 |
| **9** | 37.50_379 | 13-methylnonacosane | 2930 | 2927 | 86±12 | 34±7 | 2.51 | 0.82 | 27±5 | 3±0.7 | 8.15 | 0.14 |
| **10** | 39.64_239 | Triacontane | 3000 | 3003 | 104±10 | 157±40 | 0.66 | 41.00 | 169±23 | 101±6 | 1.67 | 1.10 |
| **11** | 41.22_435 | Hentriacontane | 3100 | 3117 | 235±63 | 67±5 | 3.52 | 1.80 | 57±8 | 27±5 | 2.15 | 0.58 |
| **12** | 41.53_436 | 2-Methylhentriacontane | 3162 | 3152 | 213±57 | 68±1 | 3.13 | 2.30 | 36±10 | 16±2 | 2.23 | 7.40 |
| **13** | 42.01_424 | 3-Methylhentriacontane | 3172 | 3182 | 69±18 | 34±10 | 2.06 | 13.00 | 18±1 | 6±1 | 3.11 | 0.0027 |
| **14** | 42.37_449 | Dotriacontane | 3200 | 3203 | 750±130 | 266±33 | 2.82 | 0.36 | 208±37 | 72±10 | 2.89 | 0.36 |
| **15** | 42.50_477 | 10-Methyldotriacontane | 3235 | 3218 | 1001±75 | 541±36 | 1.85 | 0.033 | 287±51 | 139±32 | 2.07 | 2.70 |
| **16** | 42.60_450 | 8-Methyldotriacontane | 3240 | 3221 | 297±37 | 88±9 | 3.39 | 0.0031 | 47±5 | 16±3 | 2.96 | 0.026 |
| **17** | 42.78_464 | Unknown4 | - | 3231 | 66±6 | 19±3 | 3.53 | 0.0006 | 11±1 | 3±1 | 3.57 | 0.0077 |
| **18** | 42.87_466 | Unknown5 | - | 3237 | 261±8 | 96±9 | 2.71 | 0.0001 | 57±12 | 20±3 | 2.86 | 0.89 |
| **19** | 42.96_478 | Unknown6 | - | 3249 | 204±13 | 61±7 | 3.34 | 0.0012 | 37±7 | 15±2 | 2.44 | 1.10 |
| **20** | 43.22_481 | 2-Methyldotriacontane | 3263 | 3266 | 406±49 | 242±21 | 1.67 | 8.90 | 85±9 | 37±8 | 2.21 | 0.25 |
| **21** | 43.40_416 | Unknown7 | - | 3276 | 435±86 | 240±16 | 1.81 | 0.67 | 83±6 | 38±7 | 2.17 | 0.056 |
| **22** | 44.03_463 | 15-Methyltritriacontane | 3333 | 3323 | 435±86 | 242±44 | 1.80 | 7.00 | 84±8 | 30±5 | 2.84 | 0.032 |
| **23** | 44.47_479 | Unknown8 | - | 3351 | 951±87 | 443±49 | 2.15 | 0.054 | 159±16 | 61±14 | 2.59 | 0.10 |
| **24** | 44.88_492 | Tetratriacontane | 3400 | 3387 | 134±5 | 56±4 | 2.40 | 0.0003 | 25±1 | 9±2 | 2.82 | 0.015 |

The list consists of only the compounds that were identified; some compounds may present on the GC-MS chromatogram but are not presented due to the lack of the identification; ID includes the retention time and mass to charge ratio that used to identify the chemical; “Unknown” features are compounds that were identified as hydrocarbons by NIST database, but were reported as unknown because of the big difference between the NIST RI and the calculated RI (> 30 difference); RT=retention time; NIST RI=retention indices acquired from National Institute of Standards and Technology database (NIST); Calculated RI=retention indices calculated using n-alkane standard C7-C40; Fold changes obtained from dividing GC-MS response (areas) of resistant insects by the GC-MS response (areas) of susceptible insects; p values were generated by t-tests as ($p \le 0.05$, n=4); ND=not detected.
